# The Relationship between HERV, Interleukin, and Transcription Factor Expression in ZIKV Infected versus Uninfected Trophoblastic Cells

**DOI:** 10.3390/cells13171491

**Published:** 2024-09-05

**Authors:** Anderson Luís da Costa, Paula Prieto-Oliveira, Márcia Duarte-Barbosa, Robert Andreata-Santos, Cristina M. Peter, Thamires Prolo de Brito, Fernando Antoneli, Ricardo Durães-Carvalho, Marcelo R. S. Briones, Juliana T. Maricato, Paolo M. A. Zanotto, Denis Jacob Machado, Luiz M. R. Janini

**Affiliations:** 1 Laboratory of Retrovirology, Discipline of Infectology, Department of Medicine, Federal University of São Paulo (EPM-UNIFESP), São Paulo 04039-032, Brazil; andersonstaphy@gmail.com (A.L.d.C.); mardbarbs@gmail.com (M.D.-B.); 2 Department of Bioinformatics and Genomics, College of Computing and Informatics, University of North Carolina at Charlotte, 9331 Robert D. Snyder Rd., Charlotte, NC 28223, USA; polivei1@charlotte.edu (P.P.-O.); dmachado@charlotte.edu (D.J.M.); 3 Computational Intelligence to Predict Health and Environmental Risks Center, University of North Carolina at Charlotte, 9201 University City BLVD, Charlotte, NC 28223, USA; 4 Laboratory of Retrovirology, Discipline of Microbiology, Department of Microbiology, Immunology and Parasitology, Federal University of São Paulo, São Paulo 04039-032, Brazil; randreatas@gmail.com (R.A.-S.); cristinamendespeter@gmail.com (C.M.P.); thamires.prolo@unifesp.br (T.P.d.B.); rdcarval@gmail.com (R.D.-C.); juliana.maricato@unifesp.br (J.T.M.); 5 Center for Medical Bioinformatics, Federal University of São Paulo, São Paulo 04039-032, Brazil; fernando.antoneli@unifesp.br (F.A.); marcelo.briones@unifesp.br (M.R.S.B.); 6 Department of Morphology and Genetics, Federal University of São Paulo, São Paulo 04039-032, Brazil; 7 Laboratory of Molecular Evolution and Bioinformatics, Department of Microbiology, Institute of Biosciences, University of São Paulo, São Paulo 05508-000, Brazil; pzanotto@usp.br

**Keywords:** ZIKV, interleukins, transcription factors, ERV, trophoblast cells

## Abstract

Zika virus (ZIKV) is an arbovirus with maternal, sexual, and TORCH-related transmission capabilities. After 2015, Brazil had the highest number of ZIVK-infected pregnant women who lost their babies or delivered them with Congenital ZIKV Syndrome (CZS). ZIKV triggers an immune defense in the placenta. This immune response counts with the participation of interleukins and transcription factors. Additionally, it has the potential involvement of human endogenous retroviruses (HERVS). Interleukins are immune response regulators that aid immune tolerance and support syncytial structure development in the placenta, where syncytin receptors facilitate vital cell-to-cell fusion events. HERVs are remnants of ancient viral infections that integrate into the genome and produce syncytin proteins crucial for placental development. Since ZIKV can infect trophoblast cells, we analyzed the relationship between ZIKV infection, HERV, interleukin, and transcription factor modulations in the placenta. To investigate the impact of ZIKV on trophoblast cells, we examined two cell types (BeWo and HTR8) infected with ZIKV-MR766 (African) and ZIKV-IEC-Paraíba (Asian–Brazilian) using Taqman and RT2 Profiler PCR Array assays. Our results indicate that early ZIKV infection (24–72 h) does not induce differential interleukins, transcription factors, and HERV expression. However, we show that the expression of a few of these host defense genes appears to be linked independently of ZIKV infection. Future studies involving additional trophoblastic cell lineages and extended infection timelines will illuminate the dynamic interplay between ZIKV, HERVs, interleukins, and transcription factors in the placenta.

## 1. Introduction

The *Orthoflavivirus zikaense* (class *Flasuviricetes*; order *Amarillovirales*; family *Flaviviridae*; genus *Orthoflavivirus*; Zika virus or ZIKV) [1,2,3] is a single-stranded positive-sense RNA virus whose genome is arranged in a linear, no segmented configuration with two flanking noncoding regions: 5′ NCR and 3′ NCR [4]. Viral open reading frame (ORF) codes for a polyprotein that can be cleaved into ten proteins. Three are structural proteins: capsid (C), pre-membrane/membrane (PrM), and envelope (E). The remaining seven are nonstructural proteins: NS1, NS2A, NS2B, NS3, NS4A, NS4B, and NS5 [4,5,6,7]. Historically, the Zika virus was named based on the locality of the initial isolation in Zika Forest in Uganda, Africa. This African isolate, also known as MR766, was identified in 1947 from rhesus monkey serum during surveillance for YFV (Species: *Orthoflavivirus flavi*) [7]. Phylogenetically, ZIKV can be divided into two lineages: African and Asian. These lineages may be associated with differences in virulence. African and Asian lineages are phenotypically different due to mutations acquired during viral evolution [5].

ZIKV has been sporadically detected in *Aedes aegypti* mosquitoes from Africa and Asia [4,7]. A new lineage that emerged from Asia in the 1960s and again in the 2010s has caused outbreaks in Southeast Asia, several Pacific Islands, and the Americas. In Brazil, for example, ZIKV has been identified as the etiologic agent behind the outbreak of an illness with symptoms of rash, mild fever, and arthralgia between 2014 and 2015 [8,9]. Moreover, during the Brazilian epidemic, it was observed that ZIKV could cross the maternal–fetal barrier during pregnancy and cause severe problems to the infant, such as neuronal death, abnormal vasculature, including leaky blood–brain barrier, cell cycle arrest, and apoptosis of neural progenitor cells. These problems result in postnatal microcephaly with brain damage, which was later described as Congenital ZIKV Syndrome (CZS) [9,10].

The placenta is a transient organ involved with reproduction, connecting mother and fetus to confer nutrition, oxygenation, and immune protection for the fetus [11]. While the Asian lineage has been linked to CZS, studies indicated that the African isolate may be associated with stronger virulence, higher viral replication, and lytic infections of placental cells [12]. These characteristics may lead to an early termination of pregnancy, while the Asian isolated can be less destructive to placental cells, allowing the pregnancy to proceed but interfering with the embryo development [4,10,13].

The placenta is a heterogeneous organ formed by different tissues and can support the expression of Endogenous Retroviruses (ERVs) [14]. Human ERVs represent around 8% of the human genome and are found in various vertebrate species [15,16]. These ERVs may have resulted from an ancient retroviral infection that integrated into the host genome, especially in the germline, and became endogenous [16]. Due to mutation accumulation, most ERVs are inactive, but some can be expressed [17,18,19]. Furthermore, they are regulated by DNA methylation [20,21] and are active mainly in the testis and placenta [19]. Some ERV families are involved in the placentation process through the syncytiotrophoblast formation by trophoblastic cell fusion [14].

In humans, *ERVW-1* and *ERVFRD-1* are the HERVs that produce functional proteins during placentation [22,23]. The *env* genes *ERVW-1* and *ERVFRD-1* encode for syncytin-1 and syncytin-2 proteins, respectively [22,23], which are conserved and perform essential functions in the placentation process, promoting the placental syncytiotrophoblast development. On the one hand, syncytin-1 is a glycoprotein involved in the fusion of trophoblast cells that occurs by interactions between syncytin-1 and its *SLC1A5* receptor (a neutral amino acid transporter and type D mammalian retrovirus receptor) [22]. On the other hand, syncytin-2 also has fusogenic activity, and its receptor is a member of the carbohydrate transporter superfamily MFSD2 (major facilitator superfamily domain containing 2), called *MFSD2A* [24].

Pregnancy is a complex immunological state, and shifts occur in the T helper cell (CD4^+^) balance: Th1 and Th2 [25,26]. T helper cells form a subset of T cells according to cytokine production. T helper 1 (Th1) secrete proinflammatory cytokines such as Interferon-gamma (IFN-y), whereas the T helper 2 (Th2) secrete anti-inflammatory cytokines such as IL-4 and IL-10 [27,28]. In normal conditions, down-regulation of the Th1 response and up-regulation of the Th2 response occurs during pregnancy to induce maternal tolerance and suppression [25,26]. HERVs also have immunomodulatory activity during pregnancy. For example, syncytin-1 protein is involved in the placentation maintenance by inhibition of Th1 cytokines (TNF-α and IFN-γ) and chemokine CXCL10 in human blood cells [29]. Furthermore, syncytin-2 has an immunosuppressive characteristic and plays a role in maternal–fetal immune tolerance during pregnancy [30].

Th1 and Th2 cells arise from the same precursor naïve T lymphocytes [31,32]. IFN-γ determines the differentiation of these cells in Th1 lymphocytes [31], whereas IL4 induces the differentiation of naïve T cells in Th2 lymphocytes [32].

Viral infections may activate antiviral and inflammatory pathways [33]. The host immune response plays a vital role in the clinical course of patients with viral infection, cellular immunity, and components of the innate immune response, such as interferons and other cytokines, which play an essential role in viral control [33]. ZIKV can induce an intense immune response in the developing fetus, with probable involvement of ERVs [34,35]. Ponferrada et al. (2003) observed that *ERVW-1* has a role in the host defense against retroviruses [36]. However, the regulatory mechanisms and expression of the ERVs due to the Zika infection in the placental environment are still unknown.

In the current study, we aimed to observe if the infection of placental cell lineages by two different ZIKV isolates can modulate the expression of HERVs and host defense genes (including interleukins and transcription factors) compared to controls without infection. In addition, we tested if the modulation of HERVs could be correlated with the modulation of the group of genes involved with the host defense. Furthermore, previous studies on the modulation of host defense genes in the placenta focused on the late stages of infection or postpartum [37]. Therefore, we focus on producing information in the early stages of ZIKV infection (from 24 to 72 h post-infection).

## 2. Materials and Methods

### 2.1. Cells, Viruses, and Infections

Two cell types were chosen based on their availability (they were available for the study and could be used) and unique characteristics to consider the effects on different cell lines. BeWo CCL-98™ (ATCC^®^) is derived from human choriocarcinoma [38]. HTR-8/SVneo CRL-3271™ (ATCC^®^) is derived from human trophoblasts [39]. These are placental cell lines and were kindly provided by Dr. Estela Bevilacqua from the Institute of Biomedical Sciences, University of São Paulo, Brazil.

We reproduced the three time points that yielded the highest signal of viral particle production in the monolayer: 24 h, 48 h, and 72 h (Supplementary Materials, Figures S2–S5), according to the results from viral kinetics previously published [12]. This study evaluated seven time points: 2 h, 24 h, 48 h, 72 h, 96 h, 120 h, and 144 h.

After standardizing the curves for viral isolate quantification, viral kinetics were performed with both isolates to select the best infection times for the next step of the project, namely, for the detection of placental HERV expression and transcription factors and interleukins associated with the immune system.

For the infection, 90% confluent BeWo and HTR-8 T175 flasks were exposed to MR766 low-passage (African lineage, with a few passages in mice; GenBank: AY632535.2) and IEC-Paraíba (Asian lineage isolated by Institute Evandro Chagas; GenBank: KX2800260) ZIKV lineages. For the low-passage Zika virus MR766, a MOI of 1.0 was used, and for the Zika virus IEC, a MOI of 0.5 was used.

After one hour of incubation, the cell media was supplemented with 2% FBS, and the flasks were incubated at 37 °C with 5% CO_2_ until harvest. Cell supernatants and monolayers were separated and kept at −80 °C in preparation for microarray and Taqman^®^ assays.

### 2.2. Nucleic Acid Extraction

RNA was extracted using the Qiagen Real-Time PCR for RT2 RNA QC PCR Array column extraction kit (Qiagen Inc., Germantown, MD 20874, USA, cat.#330291), following the manufacturer’s protocol for column purification with the AllPrep DNA/RNA/Protein Mini Kit (Qiagen Inc., Germantown, MD, USA, cat.#80004). The integrity of the extracted RNA was assessed on the 2100 Bioanalyzer Instrument (Agilent Technologies Inc., Santa Clara, CA, USA) by comparing the ratio between the quantities of ribosomal RNA 28S and 18S. Nucleic acids were quantified using a NanoDrop^®^ 2000 (Thermo Fisher Scientific Inc., Wilmington, DE, USA) spectrophotometer at 260 nm and normalized to an average RNA concentration value of 200 ng/µL.

### 2.3. Molecular Detection

RNA extraction was performed using 250 µL of each time point supernatant. For viral detection in RT-PCR, the AgPath-ID™ One-Step RT-PCR Reagent kit (Thermo Fisher Scientific Inc., Carlsbad, CA, USA, cat.#4387391) was used. A total of 5 μL of RNA, extracted from each condition, was combined with 5.7 μL of RNase-free water, 3.5 μL of Buffer, 0.3 μL of enzyme, and 1.5 μL of each primer/probe. The primers/probes used were ZIKV 835 10 μM and ZIKV 1086 10 μM [40]. The readings were performed using a 7500 Real-time PCR System (Applied Biosystems, Foster City, CA 94404, USA) with the following cycling conditions: 10 min incubation at 45 °C for cDNA synthesis, 10 min at 95 °C for reverse transcriptase inactivation, and 40 cycles of 15 s at 95 °C and 45 s at 60 °C for annealing and extension. The TaqMan^®^ primers with probes selected for experimentation are described in [40] and listed in Supplementary Materials, Table S1. The primers target Zika virus envelope gene ZIKV-MR766 835 sense primer (position 835-857), with the sequence TTG GTC ATG ATA CTG CTG CTG ATT GC, and ZIKV 911c antisense primer (position 911-890), with the sequence CCT TCC ACA AAG TCC CTA TTG C.

### 2.4. Detection of ERV Expression in Placenta Cell Line

Supplementary Materials, Table S2, lists all the analyzed ERVs and the corresponding literature used to justify their inclusion in this experiment. The cDNAs obtained were quantified using NanoDrop^®^ 2000 (Thermo Fisher Scientific Inc., Wilmington, DE, USA), and the values in ng/μL for the ratio of absorbances measured at 260 nm and 280 nm were obtained and standardized to the same input of 350 ng/mL. The manufacturer’s protocol TaqMan^®^ Fast Advanced Master Mix for qPCR (Thermo Fisher Scientific Inc., Carlsbad, CA, USA, cat.#4444556) was used with some adaptations to prepare the reaction. For each reaction, 5.0 μL of TaqMan^®^ Fast Advanced Master Mix, 0.5 μL of 20× primers/probes, 3.5 μL of DEPC-Treated water (Thermo Fisher Scientific Inc., Carlsbad, CA, USA, cat.#AM9920), and 1.0 μL of cDNA were added. The material was distributed in 96-well microplates Axygen^®^ (Corning, Glendale, AZ, USA cat.# 12799438), sealed with optical adhesive (Sarstedt Sarstedtstraße 1, Nümbrecht, Germany, cat.# 951994), and subjected to the 7500 Real-time PCR System. For annealing and extension steps, the equipment was programmed to reach 50 °C for 2 min, followed by 95 °C for 10 min, and then 40 cycles of 15 s at 95 °C and 60 °C for 1 min.

### 2.5. Expression of Interleukins and Transcription Factors Related to the Immune Response

The expression of interleukins and transcription factors related to the immune response was detected using custom microarrays Qiagen Human RT2 RNA QC PCR Array (Qiagen Inc., Germantown, MD, USA, cat#330291). This system allows the analysis of the expression profile of 20 genes encoding regulatory enzymes (Supplementary Materials, Table S3). The assay includes two reference genes used as controls for the experiment: Glyceraldehyde-3-Phosphate Dehydrogenase (*GAPDH*) and RNA, 18S ribosomal 1 (*RNA18S1*). The test also contains controls for reverse transcription, positive amplification controls, and genomic DNA contamination control.

To detect the targets (controls) mentioned in Supplementary Materials, Table S4, concentrations of 800 ng/mL of cDNA per reaction were used. The protocol employed was the RT2 RNA QC PCR Array Handbook provided by the manufacturer, with some adaptations. In summary, each reaction consisted of 12.5 μL of 2× RT2 SyberGreen Master Mix, 11.5 μL of RNase-free water, and 1 μL of cDNA. For the plate quality control (RTC), 12.5 μL of 2× RT2 SyberGreen Master Mix, 11.5 μL of RNase-free water, and 1 μL of cDNA with a 1:100 dilution were used. For the quality control, standards PPC and GDC, 12.5 μL of 2× RT2 SyberGreen Master Mix and 12.5 μL of RNase-free water were used. The reactions were dispensed into the corresponding wells, with a final volume of 25 μL per well. The plate was processed in the 7500 Real-time PCR System with the following program: 2 min at 50 °C, 95 °C for 10 min for Hot-Start DNA Taq Polymerase activation, 40 cycles of 15 s at 95 °C followed by 1 min at 60 °C for fluorescence data acquisition.

### 2.6. Data Analysis

A comparative analysis of gene expression levels between the ZIKV-IEC and ZIKV-BEWO conditions was conducted to determine if we could combine the results from both lineages in our statistical experiments. To that end, we used data described in Supplementary Materials, Figures S2–S5, tab “Complete ∆∆CT values”, to conduct a paired *t*-test, a statistical method suitable for analyzing paired data where the same genes are measured under both conditions. The test yielded a t-statistic of −0.559 and a *p*-value of 0.582, indicating that the difference in expression levels between the two conditions is not statistically significant. This suggests that the observed variations in gene expression between ZIK-IEC and ZIK-MRT766 under the conditions tested and despite different MOIs for each variant are likely due to random chance rather than a true difference in biological effect. Therefore, no substantial evidence was found to support a significant difference in gene expression between these conditions in the analyzed dataset. Further statistical analyses discussed elsewhere in the manuscript are based on this evidence.

The study quantified the expression levels of three HERV genes (*ERV3-1*, *ERVFRD-1*, and *ERVW-1*), a syncytin-1 receptor (*SLC1A5*), and a syncytin-2 receptor (*MFSD2A*) using a Taqman Gene Expression Assays (Thermo Fisher, Waltham, MA, USA), as determined by their cycle threshold (CT) values. Additionally, CT values for 14 interleukins *(IFNA1*, *IFNB1*, *IFNG*, *IL10*, *IL12A*, *IL17A*, *IL1B*, *IL23A*, *IL33*, *IL4*, *IL5*, *IL6*, *TBX21*, and *TGFB1*) and five transcription factors (*FOXP3*, *GATA3*, *RORC*, *STAT1*, and *STAT3*) were obtained through PCR array. The functions of the interleukins and transcription factors tested in our study are in Supplementary Materials, Table S5. The glyceraldehyde-3-phosphate dehydrogenase (*GAPDH*) gene was selected as the reference control.

Data analysis was performed according to the manufacturer’s recommendations, with some modifications. Triplicate measurements of CT values were acquired for HERVs, and their average was utilized for subsequent analyses. ΔCT values of HERVs and interleukins were computed as the discrepancy between the target genes and the control gene whenever both CT values were available (Supplementary Materials, Table S6). ΔΔCT values were calculated as the difference between the ΔCT of infected and non-infected cells unless missing data precluded this calculation (Supplementary Materials, Table S6).

Comparisons of ΔCT values between two conditions (infected and non-infected) for each gene were performed using the Wilcoxon test. Relative quantification of gene expressions was accomplished by applying the 2^−ΔΔCT^ equation, as outlined by Livak and Schmittgen (2001) [41]. Pairwise analyses of gene expression involved Pearson’s test to compare ΔΔCT values for each gene pair (Supplementary Materials, correlation.R). Data visualization was conducted using original scripts in R version 4.3.1 (Supplementary Materials, boxplot.R) and Python version 3.9.6 (Supplementary Materials, scatterplot.py and heatmap.py). The resume of our methodology is illustrated in Supplementary Materials, Figure S1.

## 3. Results

### 3.1. Differential Expression of Host Defense Genes in Trophoblastic Cells in Response to ZIKV Infection

**Figure 1:**
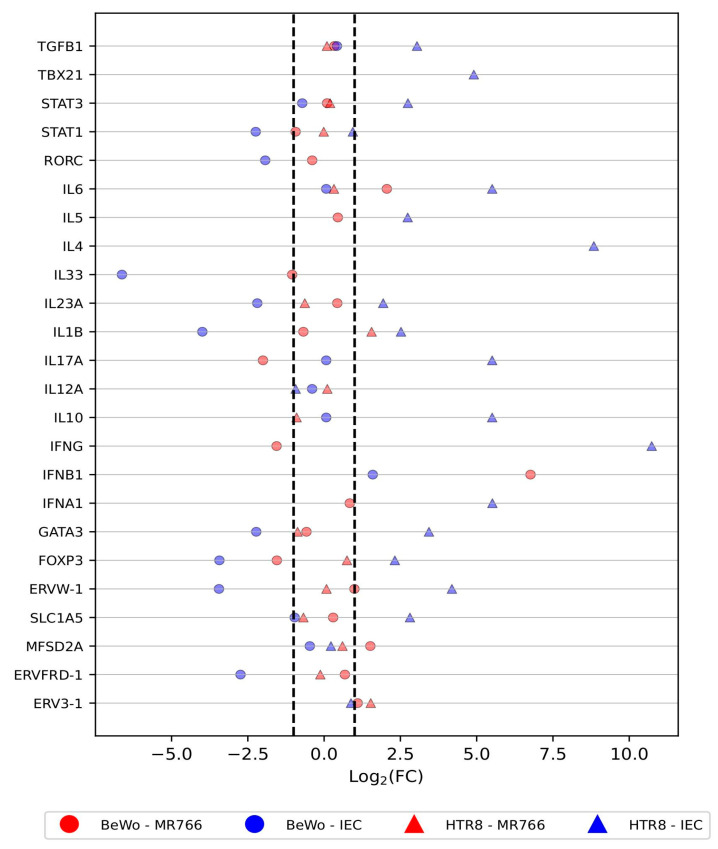
Scatterplot. Differential expression in infected and non-infected cells by Log_2_(FC). Circles: BeWo cell lineage. Triangles: HTR8 cell lineage. Red: ZIKV MR766. Blue: ZIKV IEC. The dotted lines indicate the −1 and +1 intervals.

First, we inquired if any of the selected HERVs, interleukins, or transcription factor genes would be differentially expressed in BeWo and HTR8 cells after 24 to 72 h of infection. The scatterplot (Figure 1 and Supplementary Materials, Table S7) shows the fold change of each gene in BeWo and HTR8 cell lineages under two conditions: infected or not infected by ZIKV. While most genes showed no significant change in expression upon infection, a few patterns emerged: *TBX21* and *IL-4* were slightly under-expressed in infected cells, while *IL-33* was slightly over-expressed. In contrast, genes in IEC-infected cells showed a tendency towards up-regulation, with only *STAT1*, *IL12A*, *MFSD2A*, and *ERV3-1* showing minimal change.

**Figure 2:**
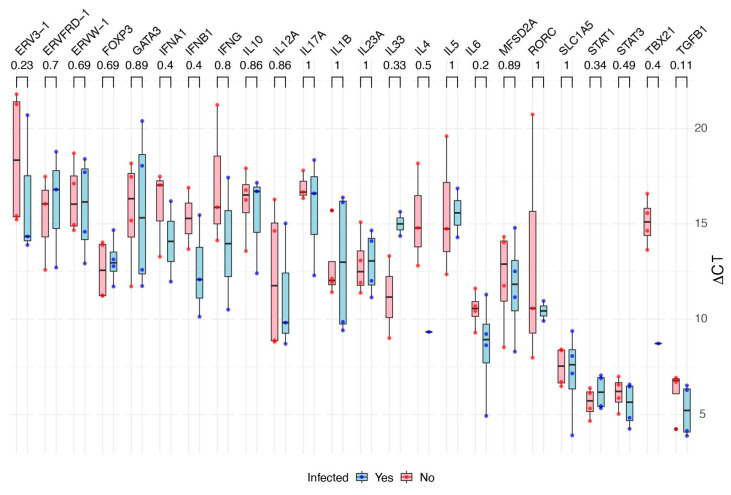
Comparison of gene ∆CTs between infected and non-infected cells. Data from different cells were grouped by gene. The blue color represents the ∆CT of each gene in infected cells, and the pink color in non-infected cells. The *p*-values from Pearson’s tests are indicated right below the gene names.

Second, we tested whether the ∆CT values of different genes would differ between infected and uninfected cells independently from their cell lineage. There was no statistical difference between each gene’s ∆CT values from infected and non-infected cells, although there were Log2(FC) values smaller than −1 or greater than +1 (Figure 2 and Supplementary Materials, Table S8).

We analyzed the data presented in Figure 2 (“Comparison of gene ∆CTs between infected and non-infected cells”) for BeWo and HTR8 together. This analysis did not reveal any statistically significant differences in gene expression within each cell line. This finding suggests that the lack of differential expression observed in the combined analysis is consistent across both cell lines and is not due to one cell line’s effect masking the other.

### 3.2. Correlation between the Expression Profiles of Selected Host Defense Genes

**Figure 3:**
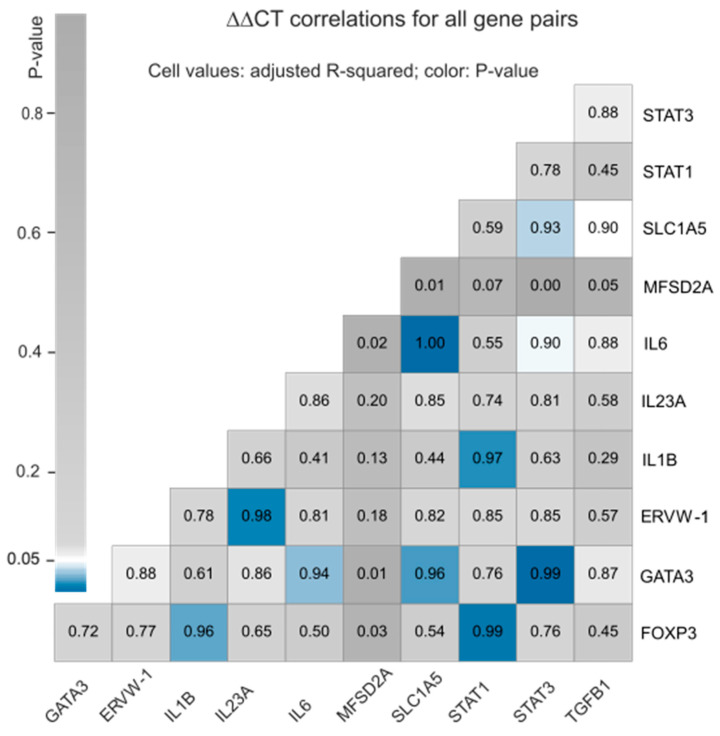
Pairwise ∆∆CT correlations. Cells contain the adjusted-R^2^ values and are colored according to the *p*-value of each correlation test. Values in the X and Y axes correspond to the different gene names.

We also investigated if different HERVs, interleukins, or transcription factors present positive or negative correlations in the selected cell lineages with or without ZIKV infection. For that purpose, we calculated the ∆∆CT values for one HERV (ERVW-1), two syncytin receptors (*MFSD2A* and *SLC1A5*), four interleukins (*IL1B*, *IL23A*, *IL6*, and *TGFB1*), and four transcription factors (*FOXP3*, *GATA3*, *STAT1*, and *STAT3*). Detailed results are listed in Supplementary Materials, Tables S8–S10. The ∆∆CT values were not computed for 13 HERVs and defensive genes, for which we did not have all the required expression data points. Four gene pairs were correlated with *p*-value < 0.01 and adjusted-R^2^ greater than 98%: *SLC1A5* vs. *IL-6*, *GATA3* vs. *STAT3*, *FOXP3* vs. *STAT1*, and *ERVW-1* vs. *IL-23A*. Another five had *p*-value < 0.05 and adjusted-R^2^ < 97%: *IL1B* vs. *STAT1*, *GATA3* vs. *SLC1A5*, *FOXP3* vs. *IL1B*, *GATA3* vs. *IL-6*, and *SLC1A5* vs. *STAT3*. These results are summarized in Figure 3.

## 4. Discussion

Barbosa et al. (2023) [12] reported data from in vitro experiments demonstrating that BeWo and HTR-8 trophoblastic cells are susceptible and permissive to two Zika virus (ZIKV) strains, ZIKV-MR766 (African or MR766) and ZIKV-IEC-Paraíba (Asian–Brazilian or IEC). Barbosa et al. (2023) [12] also demonstrated the existence of a different viral dynamic between African and Asian–Brazilian lineages in vitro. Geddes et al. (2021) [42] presented a comprehensive genome-wide transcriptome analysis of human primary astrocytes infected with Chikungunya, Mayaro, Oropouche, or Zika viruses. These authors also showed a co-evolution in the mechanisms involved in the escape of arboviruses to antiviral immune response mediated by the interferon (IFN) pathway. Castro et al. (2022) [43] quantified the modulation of HERV expression by four different encephalitic arboviruses during infection of human primary astrocytes. Their data show common HERV expression modulation by the four arboviruses, suggesting conserved evolutionary routes of transcription regulation. Furthermore, Castro et al.’s (2022) [43] results support the role of HERV induction in the transcription regulation process of genes during arboviral infections. Put together, this literature shows that trophoblastic cells are susceptive to ZIKV or other arbovirus infections, potentially affecting the expression of HERVs and interleukins. Here, we reproduced Barbosa et al.’s (2023) [12] methodology to test if these trophoblastic cell lineages would show differential expression of selected HERVs and interleukins during the early stage of ZIKV infection as suggested by previous studies such as Geddes et al. (2021) [42] and Castro et al. (2022) [43].

Our study targeted two lineages of trophoblasts derived from placental cells: BeWo cells and HTR-8. Specifically, we measured the expression of the selected ERVs, syncytin receptors, and interleukins in both cell types. We compared control cells to cells infected with two ZIKV variants, MR766 and IEC. Gene expression levels under both (infected and not-infected) conditions were measured using custom SABiosciences RT2 RNA QC PCR arrays for interleukins and Thermo Fisher’s Taqman assays for HERVs and syncytin receptors.

The Fold-Change (FC), calculated as Log_2_(2^−∆∆CT^), indicates that some selected genes could be up- or down-regulated following ZIKV infection (see Figure 1). However, the *p*-values were high and not statistically significant under a confidence interval of 95%. Moreover, as shown in Figure 2, the ∆CT profiles of different host defense genes are not statistically different when comparing infected and uninfected cells. Therefore, although certain genes may have correlated expression, we did not observe differently expressed genes in response to ZIKV infections under our experimental conditions (24 to 72 h post-infection, with MOIs between 0.5 and 1.0).

Despite the lack of response in the selected host defense genes in the early stages of ZIKV infection, we did observe a correlation between the expression profiles of several genes. We found a strong and positive correlation between the expression of nine gene pairs: *SLC1A5* and *IL6*; *GATA3* and *STAT3*; *FOXP3* and *STAT1*; *ERVW-1* and *IL23A*; *IL1B* and *STAT1*; *GATA3* and *SLC1A5*; *FOXP3* and *IL1B*; *GATA3* and *IL6*; and *SLC1A5* and *STAT3* (see Figure 3).

Put together, our observations can be explained with two congruent hypotheses. First, the target placental cells do not express the selected genes differently in response to ZIKV infection under the conditions of our experimental study. Second, the expression of those gene pairs is correlated despite ZIKV infection.

We note that fourteen genes in Figure 1 were not included in the differential expression analysis due to missing data: *IL10*, *IL17A*, *IL4*, *IL5*, *IL12A*, *IL23A*, *IL33*, *IFNA1*, *IFNB1*, *IFNG*, *RORC*, *TBX21*, *ERVFRD1*, and *ERV3-1*. Some of these genes, such as IFNA1, IFNB1, and *IFNG*, are very important for antiviral defense and might show elevated expression in response to ZIKA infection under any condition with missing data. Therefore, future studies would be wise to include those in their analysis.

It is important to highlight that the lack of differentially expressed genes in our analyses occurred in the early stage of ZIKV infection in the tested trophoblast lineages. It is necessary to contextualize our observations considering previous studies such as Geddes et al. (2021) [42], Castro et al. (2022) [43], and others that focused on different sets of ERV and interleukin genes at later time points. For example, in contrast to our study, Rabelo et al. [37] showed different expression profiles of immune response in the later stage of ZIKV infection during pregnancy in the placenta. Rabelo et al. [37] observed a release of the proinflammatory cytokines TNF and IFN-γ, which are involved in Th1 response, in human placental tissues infected by ZIKV.

Da Silva et al. [44] showed an up-regulation of IFN-γ, IFNA1, and IFNB1 in blood samples of patients infected with ZIKV during acute infection (until five days after the beginning of symptoms) in comparison with healthy subjects. The expressions of IL*6* and IL12 were similar for both groups. However, the placenta presents immunosuppressive properties [29,30,45,46,47]. Chang et al. [45] demonstrated that pretreatment with IFN-γ increased the immunosuppression performed by placenta-derived multipotent cells due to a substantial augmentation of TGFB1 expression, an anti-inflammatory cytokine. Furthermore, syncytin-1 [30] and syncytin-2 [31] are immunosuppressive proteins expressed in the placenta and inhibit the release of Th1 cytokines, especially TNF and IFN-γ. Such suppression may contribute to maternal immune tolerance.

Noteworthy, syncytin-1 has been shown to weaken the antiviral response against Influenza, leading to reduced production of IFNA1, IFNL1, and IFN-γ and inducing IL10 release by peripheral monocytes (PBMCs). However, syncytin-1 was associated with an increase in the levels of some proinflammatory cytokines (IL6 and IL1B) [48]. Additionally, up-regulation of Th2 cytokines (IL4, IL10, and IL13) and suppression of Th1 cytokines (IL2, TNF, and IFN-γ) in the feto–maternal interface contribute to a successful pregnancy and immune tolerance to the fetus [26,27,49,50]. In contrast, increased expression of Th1 and lower levels of Th2 cytokines are associated with fetal resorption and abortion [51,52].

Equally important, IL10 [53,54] is the main immunosuppressive cytokine and negatively regulates IL-12 production, which induces Th1 response and cell-mediated cytotoxicity against intracellular pathogens, such as viruses [55,56,57]. As a final point, Lu et al. [58] observed that glutamine, mediated by SLC1A5 (also called ASCT2), induced STAT3 phosphorylation and activation, causing an increase in the CCL5 expression and infiltration of T cells in oral lichen planus.

We combine our results with the cited literature to shed light on ZIKV infections in the placenta. The ZIKV epidemic that occurred in Brazil between 2015 and 2016 made clear the potential of these infections to affect the embryo/fetus, resulting in many cases of permanent neurological sequelae, including microcephaly. Despite our original expectations, we did not detect differentially expressed HERVs, syncytin receptors, interleukin, and transcription factor genes between 24 and 72 h of ZIKV infection in the two lineages of trophoblast cells, BeWo and HTR-8. Therefore, ZIKV infection did not cause correlations between pairs of gene expression profiles that we observed under our experimental conditions. We hypothesize that the suppression of immune response by the placenta may explain these findings.

Our study sheds light on ZIKV infection in trophoblastic cells, especially between the first three days of infection, but additional investigations are warranted to validate our hypothesis. For example, the data presented here pave the way for future experiments utilizing not only transcriptome but also protein-level expression. Furthermore, future research would benefit from a larger sample size that includes additional host defense genes.

## 5. Conclusions

Our study suggests two key findings. First, early infection (24–72 h) by ZIKV MR766 and IEC may not significantly alter the expression of the selected HERVs, interleukins, and transcription factors in BeWo and HTR-8 trophoblastic cell lineages. Second, even without ZIKV infection, the expression of some of these key host defense genes appears to be linked.

Different replication kinetics between strains could lead to differential impacts at later time points (which is beyond the scope of the current manuscript). This can be addressed in future experiments by extending the infection timeline and incorporating more advanced viral kinetics analyses to fully capture the potential strain-specific effects on gene expression.

While our results can be a window towards early ZIKV infection in some trophoblastic cell lineages, future studies with a larger sample size and greater diversity of host defense genes (especially those that integrate protein expression analysis), including additional trophoblastic cell lineages and more advanced time points in viral kinetics, are needed to reveal the response of these cells to ZIKV over time. We anticipate that longer infection time will trigger differential expression of ERVs, interleukins, and cytokines necessary for the formation and functioning of the placenta.

## Data Availability

The original data presented in the study are openly available in Zenodo at DOI: https://zenodo.org/doi/10.5281/zenodo.13658201 (accessed on 3 September 2024).

## Supplementary Materials

The following supporting information can be downloaded at: https://www.mdpi.com/article/10.3390/cells13171491/s1, Figure S1: Graphical abstract summarizing our main methodological steps; Figure S2: Mean BeWo infected with ZIKV-IEC. This figure shows the mean quantity of IEC viral particle production in each time point in the intracellular medium (blue) and extracellular medium (red) of BeWo cells; Figure S3: Mean BeWo infected with ZIKV-MR766Ip. This figure shows the mean quantity of MR766 viral particle production in each time point in the intracellular medium (blue) and extracellular medium (red) of BeWo cells; Figura S4: Mean HTR8 infected with ZIKV-IEC. This figure shows the mean quantity of IEC viral particle production in each time point in the intracellular medium (blue) and extracellular medium (red) of HTR8 cells; Figure S5: Mean HTR8 infected with ZIKV-MR766Ip. This figure shows the mean quantity of MR766 viral particle production in each time point in the intracellular medium (blue) and extracellular medium (red) of HTR8 cells; Table S1: Oligonucleotide primer sequences used for ERV detection for Taqman^®^ assays; Table S2: List of analyzed ERVs. Table S3: List of transcriptions factors and cytokines analyzed during array assay [14,59]; Table S4: Reference genes were used as controls for the experiment; Table S5: Classification and function of interleukins and transcription factors. Numbers correspond to the references listed below [45,60,61,62,63,64,65,66,67,68,69,70,71,72,73,74,75,76,77,78,79,80,81,82,83,84,85,86,87,88,89,90,91,92,93,94,95,96]. Table S6: This file contains four tabs: -∆CT and ∆∆CT: It contains the gene expression levels of each gene in each condition tested in our study, as well as the calculation results of the ∆CT and ∆∆CT values for each gene. -Complete ∆∆CT values: it has the ∆∆CT values of the genes that presented known expression levels for all the conditions tested in our study. -Avg. ∆CT: It contains the average ∆CTs of each gene in cells infected by IEC or MR766 and non-infected cells for the BeWo and HTR8 lineages. -Paired ∆∆CT for genes: it has the ∆∆CT values of the genes, comparing these values in four conditions: BeWo infected with MR766, BeWo infected with IEC, HTR8 infected with MR766, and HTR8 infected with IEC; Table S7: It presents the log2FC values of all the genes analyzed in our study in four conditions: BeWo infected with MR766, BeWo infected with IEC, HTR8 infected with MR766, and HTR8 infected with IEC; Table S8: It contains the expression levels of the genes evaluated in this research, as well as the ∆CT, fold change (FC), and log2FC results; Table S9: It contains the ∆∆CT values of the genes in four conditions: BeWo infected with MR766, BeWo infected with IEC, HTR8 infected with MR766, and HTR8 infected with IEC; Table S10: It is the result of the paired gene correlation analysis, which includes the R-squared and *p*-values.
