# Supplementary material for: The Relationship between HERV, Interleukin, and Transcription Factor Expression in ZIKV Infected versus Uninfected Trophoblastic Cells
Source: Cells. 2024 Sep 5;13(17):1491. doi: 10.3390/cells13171491 (PMC11394337; doi:10.3390/cells13171491)
Supplement: Supplementary file 1 [file cells-13-01491-s001.zip › supplementary Figures S1-S5 Tables S1-S5.pdf]

Table S1. Oligonucleotide primer sequences used for ERV detection for Taqman® assays.

ERV	Oligonucleotide
WE8_F (syncytin-1)	TaqManAssayThermo
WE8_R (syncytin-1)	Hs01926764_ul
FRD (syncytin-2) F	TaqManAssayThermo
FRD (syncytin-2) R	Hs01942443_sl
HR_F	TaqManAssayThermo
HR_R	Hs04184598_sl
RDR F (receptor for syncytin1)	TaqManAssayThermo
RDR R (receptor for syncytin1)	Hs01056542_ml
MFSD-2 F (receptor parasyncytin2)	TaqManAssayThermo
MFSD-2 R (receptor parasyncytin2)	Hs00293017_m1

Table S2. List of analyzed ERVs.

ERV	nmune System Funtion	Reference
H-W (syncytin-1)	Immunoregulator	Blaise *et al*. (2005) [14]
H-FRD (syncytin-2)	Immunoregulator	Blaise *et al*. (2005) [14]
H-R (ERV3-1)	Immunoregulator	angeney *et al*. (2007) [59]

REFERENCES, Table S2

14. Blaise, S.; de Parseval, N.; Heidmann, T. Functional characterization of two newly identified Human Endogenous Retrovirus coding envelope genes. *Retrovirology* **2005**, *2*, 19. doi: 10.1186/1742-4690-2-19

59. Mangeney, M.; Renard, M.; Schlecht-Louf, G.; Bouallaga, I.; Heidmann, O.; Letzelter, C.; Richaud, A.; Ducos, B.; Heidmann, T. Placental syncytins: Genetic disjunction between the fusogenic and immunosuppressive activity of retroviral envelope proteins. *Proceedings of the National Academy of Sciences* **2007**, *104*, 51, 20534-20539. doi: 10.1073/pnas.0707873105

Table S3 List of transcriptions factors and cytokines analyzed during array assay.

	Gene	Oligonucleotide
Transcription Factors Array Catalog Number 330171 CLAH25383	FOXP3 forkhead box P3	PPH00029C
	GATA3 GATA binding protein 3	PPH02143A
	RORC RAR related orphan receptor C	PPH05877A
	STAT1 Signal transducer and activator of transcription 1	PPH00811C
	STAT3 Signal transducer and activator of transcription 3	PPH00708F
Cytokines Array Catalog Number 330171 CLAH25383	IL10 interleukin 10	PPH00572C
	IL17A interleukin17a	PPH00537C
	IL6 interleukin 6	PPH00560C
	IL12A_p35 interleukin 12a	PPH00544B
	IL23A_p19 interleukin 23 α	PPH01688B
	IL33 interleukin 33	PPH17375E
	IL1B interleukin 1 beta	PPH00171C
	TGFB1 growth factor 1 beta	PPH00508A
	IL4 interleukin 4	PPH00565B
	IL5 interleukin 5	PPH00692B
	IFNA1 interferon alpha 1	PPH01321B
	IFNB1 interferon beta 1	PPH00384F
	IFNG interferon gamma	PPH00380C

Table S4. Reference genes were used as controls for the experiment.

ERV	Oligonucleotide
GAPDH (Ref.)	TaqManAssayThermo Hs03929097_g1
18SrRNA (Ref.)	TaqManAssayThermo Hs99999901_s1

Table S5: Classification and function of interleukins and transcription factors. Numbers correspond to the references listed below.

Classification	Genes	Functions
Th1 cytokines	IFNG	· Increase in recognition of antigens [1].<br>· Activation of Th1 immune response and inflammation [2].<br>· Antiviral defense: induction of NOS (nitric oxide synthase), that inhibits viral replication [3]; impediment of many stages of viral life cycle: entry, replication, gene expression, stability, release and reactivation [3].
	IL1B	· Host defense against viruses [4,5].<br>· Induction of differentiation of Th17 cells [6].
Type I interferons	IFNA1	· Rapid antiviral defense against early infection and inhibition of virus proliferation [7].
	IFNB1	· Activation of STAT1 and expression triggering of interferon-stimulated genes, with a consequent decrease in viral replication [8].
Transcription factors	STAT1	· Activation of interferons alpha and gamma [9].<br>· Antiviral defense [9,10].
	STAT3	· Proviral factor in various infections: hepatitis C virus, varicella-zoster virus, HBV, HCV, HSV-1, human CMV and measles virus [11].<br>· Antiviral role in other infections: enterovirus 71, severe acute respiratory syndrome coronavirus and human metapneumovirus [11].
	TBX21	· Stimulation of IFN-γ production and activation of antigen-specific T lymphocytes by dendritic cells. On the other hand, IFN-γ augments T-bet secretion as positive feedback [12].<br>· Reduction of Th2 (Liu [13] and Th17 responses [14,15].<br>· Development, maturation and stabilization of natural killer cells [16].
	GATA3	· Development and proliferation of natural helper cells [17].<br>· Activation of Th2 and inhibition of Th1 response [18].<br>· Development of thymocytes [19].<br>· Activation of Foxp3 expression and activity regulation of regulatory T (Treg) lymphocytes, with maintenance of immune homeostasis and immunological self-tolerance [20].
	FOXP3	· Autoregulation [21].<br>· Control of Treg cells generation, development, differentiation, function, maintenance, stability, phenotype and self-tolerance [21,22].
	RORC	· Induction of Th17 differentiation [23].<br>· Inhibition of Th1 response [23].<br>· Contribution for differentiation of innate lymphoid cells [24].
Pro- and anti-inflammatory cytokines	IL12A	· Pro-inflammatory:<br>* Induction of IFN-γ release and differentiation of Th1 cells [25].<br>* Activation of effector CD8+ lymphocytes [26].<br>* Increase in expression of T-bet [26].<br>· Anti-inflammatory: induction of IL-10 release by T cells. On the other hand, IL-10 inhibits IL-12 as negative feedback [27].

	IL33	· Pro-inflammatory:<br>* Production of Th2 cytokines and inflammation associated with them [28].<br>* Reduction of viral replication [29].<br>· Anti-inflammatory:<br>* Differentiation of alternatively activated macrophages, resulting in decreased inflammation and induction of tissue repair [30].
	TGFB1	· Inflammatory:<br>* Induction of Th17 lymphocyte differentiation [31].<br>· Anti-inflammatory:<br>* Maintenance of T lymphocyte homeostasis [32] and tolerance [31].<br>* Inhibition of CD4+ T cell response in the periphery [33].<br>* Impediment of Th1 lymphocyte differentiation [31].
Th2 cytokines	IL10	· Restriction of Th1 response [34] and IL-12 release [27].<br>· Deactivation of macrophages [35].<br>· Reduction of TNF-α and H2O2, that have antimicrobial action [35].
	IL4	· Activation of resting B lymphocytes [36] and T cells [37].<br>· Increase in proliferation of mast cells [37].<br>· Decrease in the production of IFN-α [38].

REFERENCES, Table S5

1. Gottfried-Blackmore, A.; Kaunzner, U.W.; Idoyaga, J.; Felger, J.C.; McEwen, B.S.; Bulloch, K. Acute in vivo exposure to interferon-γ enables resident brain dendritic cells to become effective antigen presenting cells. *Proc. Natl. Acad. Sci.* **2009**, *106*, 20918–20923, https://doi.org/10.1073/pnas.0911509106.
2. Bendriss-Vermare, N.; Burg, S.; Kanzler, H.; Chaperot, L.; Duhen, T.; de Bouteiller, O.; D'Agostini, M.; Bridon, J.-M.; Durand, I.; Sederstrom, J.M.; et al. Virus overrides the propensity of human CD40L-activated plasmacytoid dendritic cells to produce Th2 mediators through synergistic induction of IFN-γ and Th1 chemokine production. *J. Leukoc. Biol.* **2005**, *78*, 954–966, https://doi.org/10.1189/jlb.0704383.
3. Kang, S.; Brown, H.M.; Hwang, S. Direct Antiviral Mechanisms of Interferon-Gamma. *Immune Netw.* **2018**, *18*, e33, https://doi.org/10.4110/in.2018.18.e33.
4. Lucinda, N.; Figueiredo, M.M.; Pessoa, N.L.; Santos, B.S.d.S.; Lima, G.K.; Freitas, A.M.; Machado, A.M.V.; Kroon, E.G.; Antonelli, L.R.D.V.; Campos, M.A. Dendritic cells, macrophages, NK and CD8+ T lymphocytes play pivotal roles in controlling HSV-1 in the trigeminal ganglia by producing IL1-beta, iNOS and granzyme B. *Virol. J.* **2017**, *14*, 1–15, https://doi.org/10.1186/s12985-017-0692-x.
5. Sergerie, Y.; Rivest, S.; Boivin, G. Tumor Necrosis Factor–α and Interleukin-1β Play a Critical Role in the Resistance against Lethal Herpes Simplex Virus Encephalitis. *J. Infect. Dis.* **2007**, *196*, 853–860, https://doi.org/10.1086/520094.
6. Acosta-Rodriguez, E.V.; Napolitani, G.; Lanzavecchia, A.; Sallusto, F. Interleukins 1β and 6 but not transforming growth factor-β are essential for the differentiation of interleukin 17–producing human T helper cells. *Nat. Immunol.* **2007**, *8*, 942–949, https://doi.org/10.1038/ni1496.
7. Tsugawa, Y.; Kato, H.; Fujita, T.; Shimotohno, K.; Hijikata, M. Critical Role of Interferon-α Constitutively Produced in Human Hepatocytes in Response to RNA Virus Infection. *PLOS ONE* **2014**, *9*, e89869, https://doi.org/10.1371/journal.pone.0089869.
8. Sakuragi, S.; Liao, H.; Yajima, K.; Fujiwara, S.; Nakamura, H. Rubella Virus Triggers Type I Interferon Antiviral Response in Cultured Human Neural Cells: Involvement in the Control of Viral Gene Expression and Infectious Progeny Production. *Int. J. Mol. Sci.* **2022**, *23*, 9799, https://doi.org/10.3390/ijms23179799.
9. A Meraz, M.; White, J.; Sheehan, K.C.; A Bach, E.; Rodig, S.J.; Dighe, A.S.; Kaplan, D.H.; Riley, J.K.; Greenlund, A.C.; Campbell, D.; et al. Targeted Disruption of the Stat1 Gene in Mice Reveals Unexpected Physiologic Specificity in the JAK–STAT Signaling Pathway. *Cell* **1996**, *84*, 431–442, https://doi.org/10.1016/s0092-8674(00)81288-x.
10. E Durbin, J.; Hackenmiller, R.; Simon, M.; E Levy, D. Targeted Disruption of the Mouse Stat1 Gene Results in Compromised Innate Immunity to Viral Disease. *Cell* **1996**, *84*, 443–450, https://doi.org/10.1016/s0092-8674(00)81289-1.

11. Chang, C.; Yen, M.; Chen, Y.; Chien, C., Huang, H.; Bai, C.; Yen, B.L. Placenta-Derived Multipotent Cells Exhibit Immunosuppressive Properties That Are Enhanced in the Presence of Interferon-γ. *Stem Cells* **2006**, *24*, 2466–2477. doi: 10.1634/stemcells.2006-0071
12. Lugo-Villarino, G.; Maldonado-López, R.; Possemato, R.; Peñaranda, C.; Glimcher, L.H. T-bet is required for optimal production of IFN-γ and antigen-specific T cell activation by dendritic cells. *Proc. Natl. Acad. Sci.* **2003**, *100*, 7749–7754, https://doi.org/10.1073/pnas.1332767100.
13. Liu, X.; Tang, Z.; Zhang, Y.; Hu, J.; Li, D.; Zang, G.; Yu, Y. Lentivirally overexpressed T-bet regulates T-helper cell lineage commitment in chronic hepatitis B patients. *Mol. Med. Rep.* **2012**, *6*, 361–366, https://doi.org/10.3892/mmr.2012.905.
14. Mathur, A.N.; Chang, H.-C.; Zisoulis, D.G.; Kapur, R.; Belladonna, M.L.; Kansas, G.S.; Kaplan, M.H. T-bet is a critical determinant in the instability of the IL-17-secreting T-helper phenotype. *Blood* **2006**, *108*, 1595–1601, https://doi.org/10.1182/blood-2006-04-015016.
15. Intlekofer, A.M.; Banerjee, A.; Takemoto, N.; Gordon, S.M.; DeJong, C.S.; Shin, H.; Hunter, C.A.; Wherry, E.J.; Lindsten, T.; Reiner, S.L. Anomalous Type 17 Response to Viral Infection by CD8 $^+$ T Cells Lacking T-bet and Eomesodermin. *Science* **2008**, *321*, 408–411, https://doi.org/10.1126/science.1159806.
16. Gordon, S.M.; Chaix, J.; Rupp, L.J.; Wu, J.; Madera, S.; Sun, J.C.; Lindsten, T.; Reiner, S.L. The Transcription Factors T-bet and Eomes Control Key Checkpoints of Natural Killer Cell Maturation. *Immunity* **2012**, *36*, 55–67, https://doi.org/10.1016/j.immuni.2011.11.016.
17. Furusawa, J.-I.; Moro, K.; Motomura, Y.; Okamoto, K.; Zhu, J.; Takayanagi, H.; Kubo, M.; Koyasu, S. Critical Role of p38 and GATA3 in Natural Helper Cell Function. *J. Immunol.* **2013**, *191*, 1818–1826, https://doi.org/10.4049/jimmunol.1300379.
18. Zhu, J.; Min, B.; Hu-Li, J.; Watson, C.J.; Grinberg, A.; Wang, Q.; Killeen, N.; Urban, J.F.; Guo, L.; E Paul, W. Conditional deletion of Gata3 shows its essential function in TH1-TH2 responses. *Nat. Immunol.* **2004**, *5*, 1157–1165, https://doi.org/10.1038/ni1128.
19. Pai, S.-Y.; Truitt, M.L.; Ting, C.-N.; Leiden, J.M.; Glimcher, L.H.; Ho, I.-C. Critical Roles for Transcription Factor GATA-3 in Thymocyte Development. *Immunity* **2003**, *19*, 863–875, https://doi.org/10.1016/s1074-7613(03)00328-5.
20. Wang, Y.; Su, M.A.; Wan, Y.Y. An Essential Role of the Transcription Factor GATA-3 for the Function of Regulatory T Cells. *Immunity* **2011**, *35*, 337–348, doi:10.1016/j.immuni.2011.08.012.
21. Bending, D.; Ono, M. From stability to dynamics: understanding molecular mechanisms of regulatory T cells through*Foxp3*transcriptional dynamics. *Clin. Exp. Immunol.* **2018**, *197*, 14–23, https://doi.org/10.1111/cei.13194.
22. Kawakami, R.; Kitagawa, Y.; Chen, K.Y.; Arai, M.; Ohara, D.; Nakamura, Y.; Yasuda, K.; Osaki, M.; Mikami, N.; Lareau, C.A.; et al. Distinct Foxp3 enhancer elements coordinate development, maintenance, and function of regulatory T cells. *Immunity* **2021**, *54*, 947–961.e8, https://doi.org/10.1016/j.immuni.2021.04.005.
23. Yang, X.O.; Pappu, B.P.; Nurieva, R.; Akimzhanov, A.; Kang, H.S.; Chung, Y.; Ma, L.; Shah, B.; Panopoulos, A.D.; Schluns, K.S.; et al. T Helper 17 Lineage Differentiation Is Programmed by Orphan Nuclear Receptors RORα and RORγ. *Immunity* **2008**, *28*, 29–39, https://doi.org/10.1016/j.immuni.2007.11.016.
24. Croft, C.A.; Thaller, A.; Marie, S.; Doisne, J.-M.; Surace, L.; Yang, R.; Puel, A.; Bustamante, J.; Casanova, J.-L.; Di Santo, J.P. Notch, RORC and IL-23 signals cooperate to promote multi-lineage human innate lymphoid cell differentiation. *Nat. Commun.* **2022**, *13*, 1–14, https://doi.org/10.1038/s41467-022-32089-3.
25. A Seder, R.; Gazzinelli, R.; Sher, A.; E Paul, W. Interleukin 12 acts directly on CD4+ T cells to enhance priming for interferon gamma production and diminishes interleukin 4 inhibition of such priming. *Proc. Natl. Acad. Sci.* **1993**, *90*, 10188–10192, https://doi.org/10.1073/pnas.90.21.10188.
26. Yang, Q.; Li, G.; Zhu, Y.; Liu, L.; Chen, E.; Turnquist, H.; Zhang, X.; Finn, O.J.; Chen, X.; Lu, B. IL-33 synergizes with TCR and IL-12 signaling to promote the effector function of CD8$^+$ T cells. *Eur. J. Immunol.* **2011**, *41*, 3351–3360, https://doi.org/10.1002/eji.201141629.
27. Meyaard, L.; Hovenkamp, E.; A Otto, S.; Miedema, F. IL-12-induced IL-10 production by human T cells as a negative feedback for IL-12-induced immune responses. *J. Immunol.* **1996**, *156*, 2776–2782, https://doi.org/10.4049/jimmunol.156.8.2776.
28. Han, X.; Chai, R.; Qi, F.; Bai, S.; Cui, Y.; Teng, Y.; Liu, B. Natural Helper Cells Mediate Respiratory Syncytial Virus-Induced Airway Inflammation by Producing Type 2 Cytokines in an IL-33-Dependent Manner. *Immunotherapy* **2017**, *9*, 715–722, https://doi.org/10.2217/imt-2017-0037.
29. Gao, X.; Chi, X.; Wang, X.; Wu, R.; Xu, H.; Zhan, M.; Li, D.; Ding, Y.; Xu, D.; Niu, J. IL-33 Inhibits Hepatitis B Virus through Its Receptor ST2 in Hydrodynamic HBV Mouse Model. *Mediat. Inflamm.* **2020**, *2020*, 1–9, https://doi.org/10.1155/2020/1403163.
30. Faas, M.; Ipseiz, N.; Ackermann, J.; Culemann, S.; Grüneboom, A.; Schröder, F.; Rothe, T.; Scholtysek, C.; Eberhardt, M.; Böttcher, M.; et al. IL-33-induced metabolic reprogramming controls the differentiation of

alternatively activated macrophages and the resolution of inflammation. *Immunity* **2021**, *54*, 2531–2546.e5, https://doi.org/10.1016/j.immuni.2021.09.010.
31. Li, M.O.; Wan, Y.Y.; Flavell, R.A. T Cell-Produced Transforming Growth Factor-β1 Controls T Cell Tolerance and Regulates Th1- and Th17-Cell Differentiation. *Immunity* **2007**, *26*, 579–591, https://doi.org/10.1016/j.immuni.2007.03.014.
32. Gorelik, L.; A Flavell, R. Abrogation of TGFβ Signaling in T Cells Leads to Spontaneous T Cell Differentiation and Autoimmune Disease. *Immunity* **2000**, *12*, 171–181, https://doi.org/10.1016/s1074-7613(00)80170-3.
33. Robinson, R.T.; Gorham, J.D. TGF-β1 Regulates Antigen-Specific CD4+ T Cell Responses in the Periphery. *J. Immunol.* **2007**, *179*, 71–79, https://doi.org/10.4049/jimmunol.179.1.71.
34. Fiorentino, D.F.; Bond, M.W.; Mosmann, T.R. Two types of mouse T helper cell. IV. Th2 clones secrete a factor that inhibits cytokine production by Th1 clones. *J. Exp. Med.* **1989**, *170*, 2081–2095, https://doi.org/10.1084/jem.170.6.2081.
35. Bogdan, C.; Vodovotz, Y.; Nathan, C. Macrophage deactivation by interleukin 10. *J. Exp. Med.* **1991**, *174*, 1549–1555, https://doi.org/10.1084/jem.174.6.1549.
36. Rabin, E.M.; Ohara, J.; E Paul, W. B-cell stimulatory factor 1 activates resting B cells. *Proc. Natl. Acad. Sci.* **1985**, *82*, 2935–2939, https://doi.org/10.1073/pnas.82.9.2935.
37. Mosmann, T.R.; Sad, S. The expanding universe of T-cell subsets: Th1, Th2 and more. *Immunol. Today* **1996**, *17*, 138–146, https://doi.org/10.1016/0167-5699(96)80606-2.
38. Hober, D.; Benyoucef, S.; Chehadeh, W.; Chieux, V.; La Tribonniere, D.; Mouton, Y.; Bocket, L.; Wattre, P. Production of Interleukin-4, Interferon (IFN)-γ and IFN-α in Human Immunodeficiency Virus-1 Infection: An Imbalance of Type 1 and Type 2 Cytokines may Reduce the Synthesis of IFN-α. *Scand. J. Immunol.* **1998**, *48*, 436–442, https://doi.org/10.1046/j.1365-3083.1998.00417.x.

Figure S1. Summarized methodology.

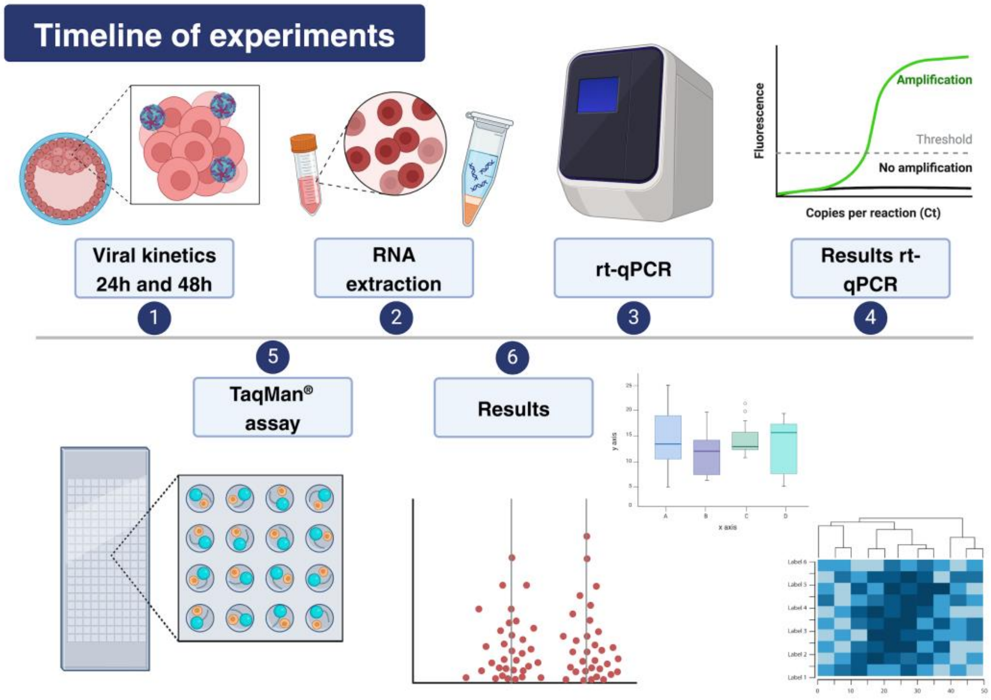

Viral Kinetics Results

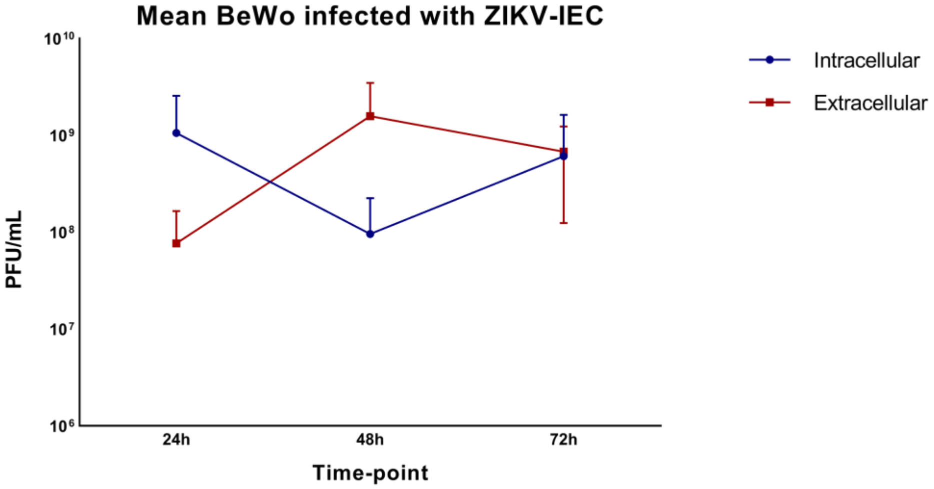

Figure S2. Mean BeWo infected with ZIKV-IEC. This figure shows the mean quantity of IEC viral particle production in each time point in the intracellular medium (blue) and extracellular medium (red) of BeWo cells.

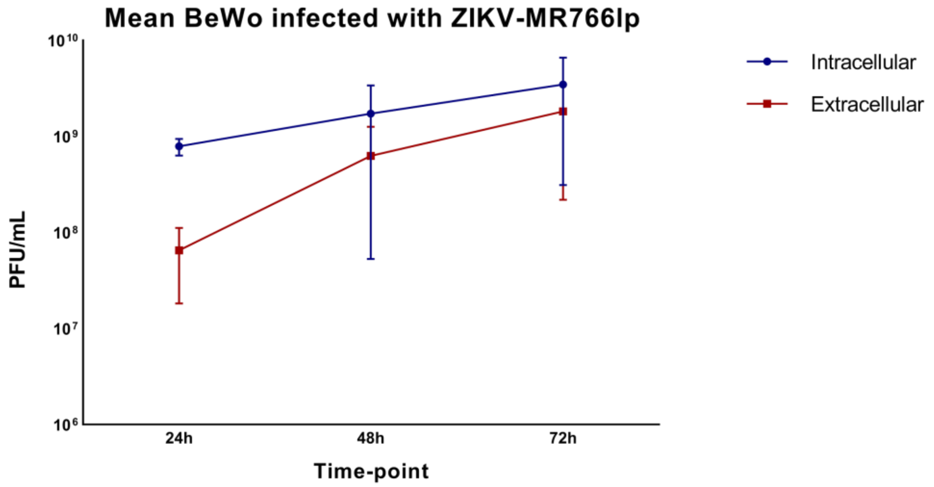

Figure S3. Mean BeWo infected with ZIKV-MR766Ip. This figure shows the mean quantity of MR766 viral particle production in each time point in the intracellular medium (blue) and extracellular medium (red) of BeWo cells.

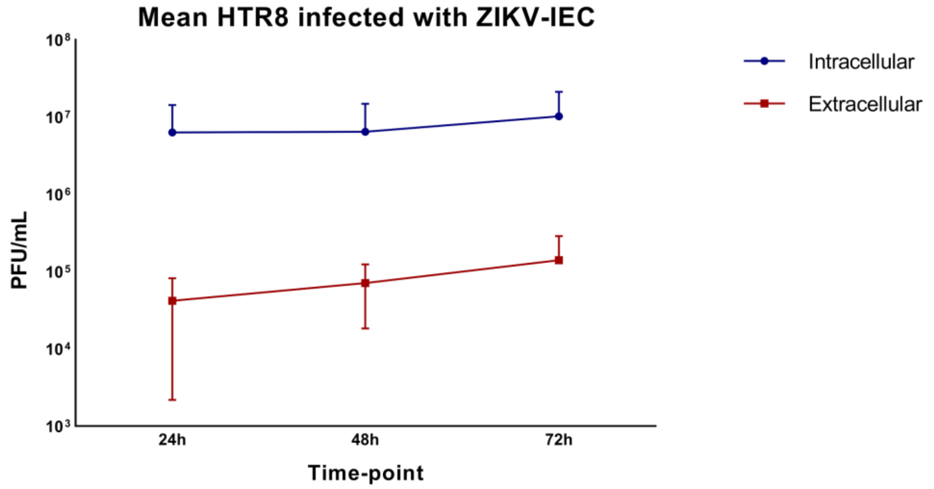

Figure S4. Mean HTR8 infected with ZIKV-IEC. This figure shows the mean quantity of IEC viral particle production in each time point in the intracellular medium (blue) and extracellular medium (red) of HTR8 cells.

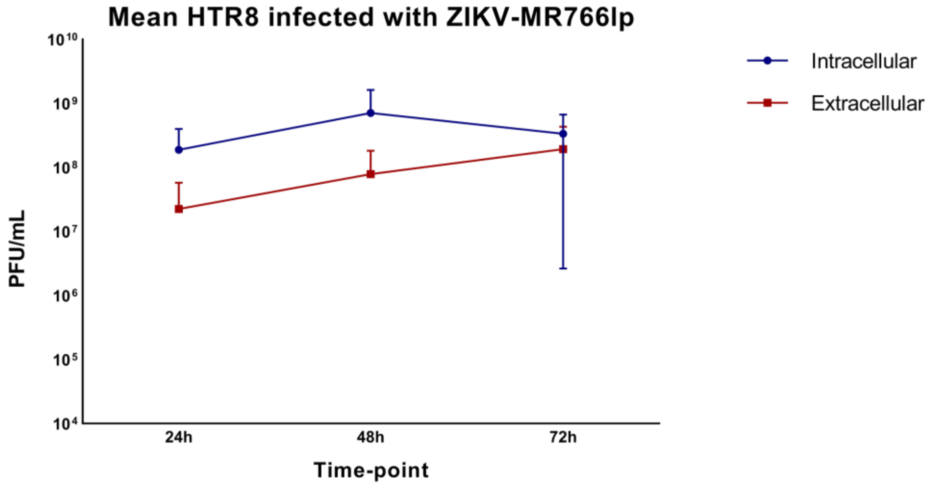

Figure S5. Mean HTR8 infected with ZIKV-MR766Ip. This figure shows the mean quantity of MR766 viral particle production in each time point in the intracellular medium (blue) and extracellular medium (red) of HTR8 cells.
